# Sodium Values During the First 10 Postnatal Days in Extremely-Low-Birth-Weight Infants and Long-Term Neurocognitive Outcomes: A Systematic Review

**DOI:** 10.3390/children13020287

**Published:** 2026-02-19

**Authors:** Sara Beyen, Karel Allegaert, Thomas Salaets, Anke Raaijmakers

**Affiliations:** 1 Department of Development and Regeneration, KU Leuven, 3000 Leuven, Belgium; sarah.beyen@student.kuleuven.be; 2 Department of Pharmaceutical and Pharmacological Sciences, KU Leuven, 3000 Leuven, Belgium; 3 Department of Hospital Pharmacy, Erasmus Medical Center, 3015 GD Rotterdam, The Netherlands; 4 Department of Cardiovascular Sciences, KU Leuven, 3000 Leuven, Belgium; thomas.1.salaets@uzleuven.be; 5 Department of Paediatric Nephrology, Sydney Children’s Hospital, Randwick, NSW 2031, Australia; anke.raaijmakers@health.nsw.gov.au; 6 School of Women’s and Children’s Health, University of New South Wales, Kensington, NSW 2031, Australia

**Keywords:** extremely low birth weight infants, sodium imbalances, hyponatremia, hypernatremia, neurodevelopmental outcomes, intraventricular hemorrhage

## Abstract

**Highlights:**

**What are the main findings?**

- Based on our systematic review, we found a possible association between hypernatremia in ELBW infants and IVH.
- Hyponatremia was associated with long-term neurocognitive outcomes (i.e., hearing loss and a trend to neurocognitive impairment).

**What are the implementations of the main findings?**

- This systematic review suggests a possible association between early sodium disturbances and adverse neurodevelopmental outcomes in ELBW infants, emphasizing the need for further high-quality, prospective studies, especially since sodium management can be modulated.

**Abstract:**

Purpose: To synthesize all existing literature on the association between sodium disturbances during the first 10 days of life in Extremely-Low-Birth-Weight (ELBW) infants and the risk of developing severe intraventricular hemorrhage (IVH > grade 1) or long-term neurodevelopmental impairment. Methods: Applying systematic review (ID CDR42024622933) principles, five major databases were explored. Any study was included if it reported on ELBW infants, on serum sodium values within the first 10 postnatal days, or was related these to neurocognitive or neurodevelopmental outcomes. Results: Ten studies (13,276 infants) met inclusion criteria. Six studies evaluated the association between hypernatremia (>145 or >150 mmol/L) and severe IVH, and two reported a significant association. Among two studies studying hyponatremia (ranging <130 or <120 mmol/L), one found a significant association with severe IVH. Evidence regarding sodium fluctuations (difference between the maximum and minimum serum sodium values) identified fluctuations >13 mmol/L as a strong risk factor for severe IVH, while another study showed that glucose-corrected sodium fluctuations were independently associated with severe IVH. Long-term neurodevelopmental outcomes were reported in four studies; hyponatremia was significantly associated with hearing loss in one study (OR 5.6 (95% CI 1.1–27.8)), while another study reported that glucose-corrected sodium fluctuations were associated with neurodevelopmental impairment at 18–21 months, although significance disappeared after adjustment for confounding factors. Conclusion: Considering the limitations related to heterogeneity in study design, threshold sodium values and cohort size, this systematic review suggests a possible association between early sodium disturbances and adverse neurodevelopmental outcomes in ELBW infants, emphasizing the need for further high-quality, prospective studies, especially since sodium management can be modulated.

## 1. Introduction

Extremely-Low-Birth-Weight (ELBW, birth weight <1000 g) infants are at increased risk of mortality and morbidity. This extends to both short- and long-term morbidities, including those affecting the central nervous system [1]. Despite marked improvements in mortality, these morbidity rates have not prominently improved [2]. Neurocognitive development is multifactorial and influenced by antenatal, perinatal, and postnatal factors [1]. Electrolyte imbalances have been proposed as a potential contributing factor. Dysnatremia, as either hyponatremia, hypernatremia or sodium fluctuations, is associated with several common complications in preterm infants [3]. Studies have also indicated that neonatal dysnatremia may influence neurocognitive outcomes, partly due to its relationship with IVH development [4,5,6]. A potential pathophysiological mechanism underlying this association involves fluid shifts, driven by osmolar changes. Neonates are particularly sensitive to such shifts due to the immaturity of their brain’s adaptive mechanisms [7]. To provide a contemporary overview on the topic, we conducted a systematic review to synthesize the data from studies examining the relationship between sodium values during the first 10 days after birth and long-term neurocognitive outcomes and IVH in ELBW infants.

## 2. Methods

### 2.1. Registration and Ethics

The protocol was registered in PROSPERO (ID CDR42024622933, 19 December 2024). Ethics approval was obtained from the Ethics Board of KU Leuven, Belgium (MP034156), on 28 November 2024.

### 2.2. Search Strategy

We conducted a comprehensive search across the following databases: PubMed, Embase, Web of Science Core Collection, Scopus, and CINAHL. The search was performed to include any study available in these databases up to 5 December 2024. No filters were applied. The search strategy was developed using predefined keywords related to ELBW infants and serum sodium levels, with expert input from an expert data scientist (Biomedical Librarian, Ms. Krizia Tuand, KU Leuven). A detailed overview of the search strategy, including the free-text terms used, is provided in Table S1. The Covidence platform was used for the collection of data and analysis [8]. This systematic review was conducted in accordance with the principles outlined by the Preferred Reporting Items for Systematic Reviews and Meta-Analyses (PRISMA) guidelines [9].

### 2.3. Study Selection Criteria

Studies were eligible for inclusion if they met the following criteria: reporting on cases or cohorts of ELBW (i.e., birth weight <1000 g) infants, with data on serum sodium values within the first 10 days of life, with subsequent reporting on neurocognitive or neurodevelopmental outcomes. Only studies published in English were included. We excluded studies involving ELBW infants with congenital conditions that could affect neurodevelopment or those with brain injury at birth.

After deduplication, two independent reviewers (S.B., K.A.) screened titles and abstracts for relevance. Full-text articles of potentially relevant studies were retrieved and were subsequently reassessed based on the selection criteria outlined and on the risk of bias and quality. In cases of disagreement, a third independent reviewer (A.R.) was consulted to solve discrepancies. Finally, the reference lists of all retained articles were checked for other relevant articles.

## 3. Results

### 3.1. Structured Literature Review

**Figure 1:**
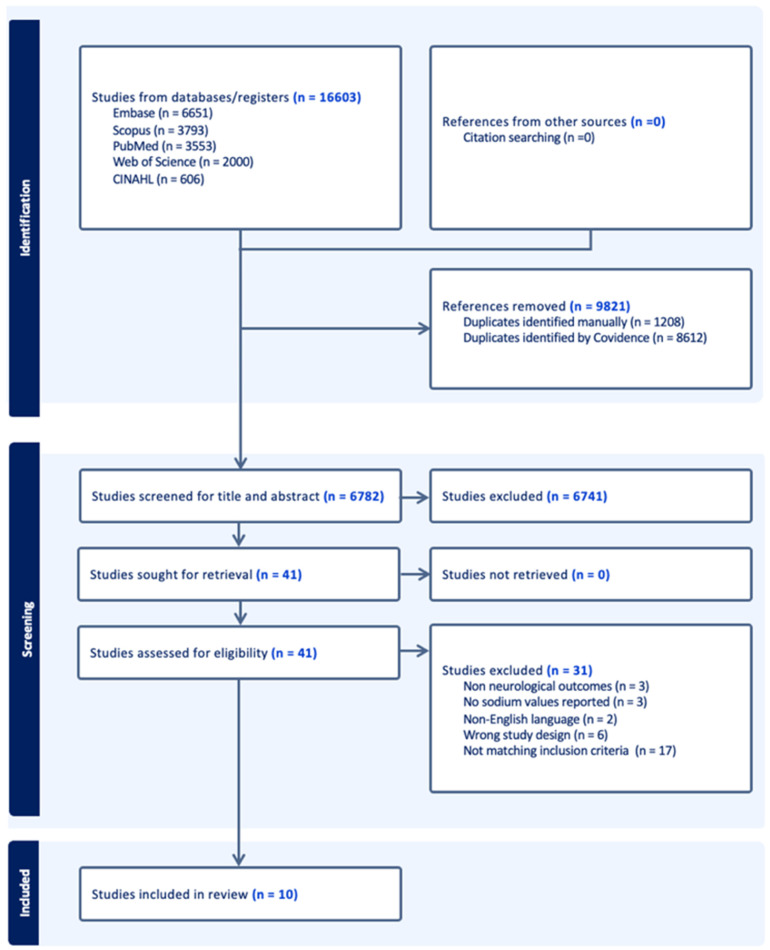
Flow diagram of structured literature review according to the PRISMA guidelines.

After the screening process, 10 studies met inclusion criteria. The PRISMA flow diagram (see Figure 1) provides a detailed overview of the selection procedure [9].

### 3.2. Baseline Characteristics

The studies included varied considerably in their design, sample size and participants’ demographics, and employed various inclusion and exclusion criteria. Table 1 summarizes the baseline characteristics of included studies. A total of 13,276 ELBW infants were analyzed across studies. The risk of bias and quality assessment is provided in the Supplementary Materials (File S1).

The mean gestational age of infants included across studies ranged from 24 to 28 weeks. The mean birth weight varied, ranging from 520 to 982 g. Data on sex ratio was available for 13,047 infants in total; 6308 (48.3%) were male and 6739 (51.7%) were female, showing a rather balanced gender distribution.

### 3.3. Exposure

Data on sodium disturbances, a summary of definitions, timing of monitoring, incidence of sodium disbalance, and trends in sodium concentrations across the studies can be found in Table 2. Exposure variables measured varied between studies.

The most common form of dysnatremia studied was hypernatremia (n = 8 studies) [3,4,6,10,11,12,13,14], followed by hyponatremia (n = 3) [3,6,11]. Serum sodium fluctuations were examined in five studies [4,5,6,14,15]. In one study, plasma sodium levels were corrected for glucose levels using the Katz formula (Na_(corr)_ = Na + 0.3 × (Glucose -5) (mmol/L)) [15]. Definitions on threshold sodium values were not standardized. Several studies also assessed different severity categories of dysnatremia. Monnikendam et al. differentiated moderate (145–154 mmol/L) from severe hypernatremia (>154 mmol/L) and moderate (125–134 mmol/L) from severe hyponatremia (<125 mmol/L) [3]. Späth et al. categorized hypernatremia into three distinct levels (>145 mmol/L, >150 mmol/L, and >155 mmol/L), allowing for nuanced analyses of severity effects [14]. Across the various cohorts of ELBW infants, the incidence of hypernatremia showed considerable variability, ranging from 5.3% to 75.7%. Hyponatremia was assessed less frequently and presented with lower incidences, ranging from 2.3% to 35.1%.

The duration of serum sodium monitoring ranged from 3 to 10 days, reflecting the postnatal age interval of interest. In all studies, the initial measurement occurred within the first 24 h of life. Sodium levels were measured at least once daily in all studies, with some studies performing measurements up to every 12 h. Four studies reported on postnatal sodium trends. Findings were consistent across three studies, showing that sodium levels typically peaked at around 48 h of life and returned to baseline between days 4 and 7 [3,4,12,15]. In contrast, Dalton et al. found that maximum serum sodium occurred later, on mean postnatal day 4 [4].

### 3.4. Outcomes

Data on short-term neurodevelopmental outcomes (severe IVH (>grade II), and long-term neurodevelopmental outcomes) can be found in Table 3. The included studies examining the association between severe IVH and sodium levels in ELBW infants consistently defined severe IVH as grade III-IV (Papile classification), detected by cranial ultrasound. The timing of IVH assessment typically ranged from day 1 to 10 of life. Although some studies did not specify exact timing, they indicated the use of routine cranial ultrasound, typically performed within this early postnatal period. Dalton et al. reported that the median day of onset was postnatal day 5 for any grade IVH, and day 4 for severe IVH [4].

The incidence of severe IVH among hypernatremic ELBW infants varied across studies. Monnikendam et al. assessed incidence between various dysnatremic subgroups. The highest incidence (28.9%) was seen in the severe hyponatremic group (<125 mEq/L), followed by the severe hypernatremic group (>154 mEq/L) (27.4%) [3]. Studies investigating the association between hypernatremia and severe IVH (n = 6) found conflicting results. Four studies reported no significant association between hypernatremia and severe IVH [4,6,12,14]. Conversely, Monnikendam et al. demonstrated a significantly higher incidence of severe IVH in infants with both moderate and severe hypernatremia [3]. Additionally, Lim et al. observed significantly higher maximal sodium levels in infants with severe IVH compared to those without severe IVH, with mean sodium concentrations in the hypernatremic range. However, this did not remain significant after multivariate analysis [5].

In contrast, studies regarding hyponatremia were very limited (number = 2). Monnikendam et al. demonstrated a significant association between both moderate and severe hyponatremia and severe IVH, whereas Lee et al. found no significant differences in hyponatremia incidence between infants with severe IVH and those without [3,6].

The role of sodium fluctuations was examined across several studies (number = 5), with varying conclusions. Two studies reported no significant associations between sodium fluctuations and severe IVH [6,14]. Similarly, Dalton et al. found no significant relationship between rapid changes in sodium levels in hypernatremic infants and the composite outcome of severe IVH or death [4]. In contrast, Lim et al. reported that changes in serum sodium of >13 mmol/L were a strong risk factor for developing severe IVH [5]. Additionally, Gervais et al. explored glucose-corrected sodium fluctuations specifically and found that these fluctuations were significantly associated with severe IVH, independent of gestational age [15].

Long-term neurodevelopmental outcomes were evaluated less frequently across studies (number = 4). Leslie et al. found hyponatremia to be significantly associated with an increased risk of SNHL, assessed at 8–10 months of corrected age [11]. This association persisted after multivariate analyses adjusting for family history for hearing impairment, maternal, and neonatal confounding factors. Finally, case reports from Saeed et al. and Sabir et al. illustrated that individual major hypernatremic (204 mmol/L and 199 mmol/L) events still resulted in subsequent normal long-term neurodevelopment [10,13].

Gervais et al. evaluated the association between sodium fluctuations (adjusted for glucose levels) and a composite neurodevelopmental impairment at 18–21 months corrected age. Initially, a significant association was found, independent of gestational age. However, this relationship lost statistical significance after adjusting for markers of neonatal illness severity (Score for Neonatal Acute Physiology) and exposure to NSAIDs used for patent ductus arteriosus treatment [15].

## 4. Discussion

Applying a systematic review, we summarized all available evidence on potential associations between dysnatremia or larger sodium fluctuations during the first 10 days of life in ELBW infants and neurocognitive outcomes, although there were inconsistent findings across studies.

### 4.1. Exploring the Mechanisms Related to Early Sodium Values and Neurodevelopment

Different direct or indirect mechanisms have been suggested, while we should remain cautious when extrapolating from association to causation. Suggested mechanisms relate to cellular swelling and osmotic changes affecting inner ear cells or neurons, hyponatremia acting as a marker of overall neonatal illness severity, or an interaction with ototoxic medications (e.g., aminoglycosides), which are themselves known hearing risk factors. The causes of hyponatremia (and ‘similar’ to hypernatremia) technically relate to sodium or fluid balance (in/out) and its modulators, including aspects like capillary leak, renal and non-renal sodium or fluid losses. Hypernatremia had been shown to cause brain shrinkage and subsequent vascular rupture with cerebral bleeding and IVH [5].

A potential pathophysiological mechanism underlying this association involves fluid shifts, driven by osmolar changes. Neonates are particularly sensitive to such shifts due to the immaturity of their brain’s adaptive mechanisms [7]. Hypernatremia has been shown to cause brain shrinkage, potentially leading to vascular rupture/strain and subsequent intracerebral hemorrhages, venous sinus thrombosis, and/or infarction. In response, the brain adapts by generating ionogenic osmoles like taurine, which increase intracellular sodium concentrations and restore water balance. However, if hypernatremia is subsequently corrected too rapidly, this adaptive response can predispose to cerebral edema [16]. Similarly, this mechanism likely also explains the fact that acute hyponatremia is a risk factor for cerebral oedema [7]. It is noteworthy that hyperglycemia often accompanies hypernatremia in preterm infants. Hyperglycemia also causes fluid shifts and has been identified as an independent risk factor for neurodevelopmental impairment [15,17]. However, dysnatremia might merely represent overall illness severity or immaturity, factors known to be strongly associated with poor neurodevelopmental outcomes. Some of our results support this statement, as the relationship between dysnatremia and neurodevelopmental outcomes lost significance after adjustment for confounding factors [5,15]. This notion is supported by Becker et al., who showed that excessive sodium supplementation, rather than fluid load, was strongly associated with overall morbidity in ELBW infants. Their findings suggest that impaired sodium homeostasis may predominantly reflect underlying clinical instability and renal immaturity [18].

### 4.2. Comparative Impact of Hyponatremia, Hypernatremia, and Sodium Fluctuations on Neurodevelopment

Among the three categories—hyponatremia, hypernatremia, and sodium fluctuations—there is no clear pattern showing which poses the greatest risk to neurodevelopment. However, some trends occur.

Hypernatremia is the most observed sodium disturbance in the first postnatal week. Several studies reported a common sodium pattern. These findings correspond closely to recent observations by van Sas et al. and Pace et al., who described sodium levels peaking on day 3, and subsequently returning to baseline by the end of the first week [19,20]. The physiological rise in sodium concentrations during the initial postnatal days reflects fluid shifts occurring predominantly within the first 24 to 48 h after birth, which are most pronounced in preterm neonates [21,22]. Despite the prevalence, its direct relationship with adverse outcomes remains debated. Only one study in our review reported a significant association between hypernatremia and severe IVH [3]; others failed to find an independent association once confounders were controlled [4,5,6,12,14].

Hyponatremia, since it is less frequently observed, has received less research attention. Monnikendam et al. found the highest incidence of severe IVH in infants with severe hyponatremia (<125 mmol/L), even higher than in those with severe hypernatremia [3]. Conversely, Lee et al. did not find any significant associations between hyponatremia and severe IVH, possibly due to small sample sizes or variable definitions [6]. However, Leslie et al. found significant associations between hyponatremia and SNHL, especially in ELBW infants [11]. While causality remains uncertain, hyponatremia may act as a surrogate marker for systemic illness or may contribute through osmotic mechanisms or ototoxic treatments like furosemide [23].

Our research proposes a possible correlation between substantial sodium fluctuations and severe IVH. Gervais et al. further demonstrated a significant association between glucose-corrected sodium variability and IVH, emphasizing the potential role of osmotic instability rather than absolute sodium values [15]. While some studies did not confirm such associations, differences in fluctuation definitions may have influenced outcomes. Importantly, these results highlight the need to define clinical thresholds.

### 4.3. Heterogeneity in Study Designs and Findings

A key finding is the marked heterogeneity in the study design, definitions, and timing of measurements. This partially explains the considerable differences observed in reported incidences among ELBW cohorts and complicates comparison and pooling of data. Pace et al. also observed this in a recent review, showing that the lack of standardized definitions represents a significant challenge for research in this field [20]. Measurement periods also varied significantly. Some studies analyzed sodium trends over 3 days, and others over 7 or 10 days. Differences in sampling windows may result in variable sensitivity for detecting associations.

The assessment of IVH was another point of inconsistency. While most studies included in our review focused on severe IVH diagnosed via cranial ultrasound, the timing of ultrasound screening and IVH detection was often imprecise. Although IVH typically occurs within the first 48–72 h of life, most studies failed to report the exact day of diagnosis. Only Dalton et al. reported a median onset of severe IVH on day 4 [4]. Without precise timing, it becomes difficult to assess causality or temporal relationships between dysnatremia and IVH onset.

Post hoc, we were somewhat surprised by the overall limited number of papers, considering the heterogeneity in type of studies and the number of participants involved (Table 1). This might be explained by the specific focus on ELBW, but is likely also because of the overall limited research in this area.

### 4.4. Major Research Gaps and Future Directions

Our review highlights several important gaps. First, there is a need for standardized definitions of hyponatremia, hypernatremia, and clinically relevant sodium fluctuations in neonatal populations. This is in line with a recent analysis on this topic, since standards or recommendations for publications that present neonatal laboratory data—including on sodium—were not identified, while published information on laboratory values for neonates is sparse, not systematic, and incomplete [24]. Such reference values or thresholds should be informed by epidemiological patterns (distribution in sodium values as observed), but even more by the link or association with clinically relevant outcome variables. Unfortunately, the volume of information retrieved does not (yet) allow the suggestion of such clinically relevant thresholds. Without consensus, it will remain difficult to compare and interpret future studies and to synthesize pooled information [24].

Second, future research should focus on temporal relationships, particularly the timing of sodium disturbances in relation to IVH onset. Prospective studies should aim to record daily sodium levels and correlate these with precisely timed cranial ultrasounds to assess causality.

Third, considering the osmolar changes as likely mechanisms, studies should correct sodium levels for glucose to correct for osmolarity. The approach by Gervais et al. of correcting sodium levels for glucose has rarely been adopted in other studies, despite the known influence of both sodium and glucose on plasma osmolality. ELBW infants frequently experience hyperglycemia due to heightened stress responses, excessive glucose supplementation, and insulin insensitivity [25]. Several studies have identified fluctuations in glucose levels as independent predictors of neurological dysfunction [25,26]. Consequently, consistently correcting plasma sodium values for glucose may prove important in future research examining the relationship between sodium concentrations and neurodevelopmental outcomes.

Finally, more research is needed linking sodium disturbances directly to long-term neurodevelopmental outcomes in the cohort of ELBW infants. Studies should incorporate standardized developmental assessments, like the Bayley Scales, and adjust for known confounders including gestational age, illness severity, and cerebral injury. This is even more relevant, since sodium fluctuations can be influenced in early neonatal life.

### 4.5. Strengths and Limitations

Our study should be interpreted with regard to its strengths and limitations. A notable strength of this systematic review is the simultaneous consideration of both severe IVH and long-term neurodevelopmental outcomes, given the potential link between these two factors. Nevertheless, our study carries certain limitations, primarily related to the retrospective nature of the studies included and the overall low number and heterogeneity of studies available for analysis. Furthermore, there is an inherent risk of confounding bias, although many studies attempted to address this through multivariate analyses. In our review, we identified numerous maternal, perinatal, and neonatal confounding variables affecting hypernatremic and hyponatremic infants. Our findings underscore the importance of adequately adjusting for these variables in future research. Additionally, most individual studies included in this review had limited sample sizes, potentially contributing to type II errors, and explaining why some studies failed to demonstrate significant associations between sodium levels and neurodevelopmental outcomes. Finally, causation cannot be established based on the existing evidence, emphasizing the necessity for larger prospective investigations. We are aware that the final list of retained papers contains large population studies, as well as case reports, while the analysis was restricted to a systematic review of the literature without meta-analysis. While this has obvious limitations, this is in line with the initial study protocol as registered in Prospero. This disabled any quantitative synthesis that was not possible, while the heterogeneity of definitions on sodium levels post hoc further complicated a quantitative synthesis. Furthermore, the case reports at least illustrate that even major sodium fluctuations do not necessarily result in neurodevelopmental impairment.

## 5. Conclusions

This systematic review suggests a potential association between early-life sodium levels in ELBW infants and later neurocognitive outcomes. Although we cannot determine whether this relationship is causal or merely reflecting underlying disease severity, these findings underscore the importance of careful early fluid and sodium management in these infants. Further research in this area is essential for both primary and secondary prevention of impaired neurocognitive outcomes.

## Figures and Tables

**Table 1:** Baseline characteristics of included studies.

Author	Publication Year	Journal	Study Type	Inclusion Criteria	Exclusion Criteria	Number of Patients (N)	Number of Cases	Number of Controls	Sex Ratio M:F	Case Sex Ratio M:F	Control Sex Ratio M:F	Mean GA ^a^ (Range) (wks)	Mean GA ^a^ (Range) Cases (wks)	Mean GA ^a^ (Range) Controls (wks)	Mean BW ^b^ (Range) (g)	Mean BW ^b^ Cases (g)	Mean BW ^b^ Controls (g)	Cases	Comparator	Quality Assessment (Tool)
M. A. Saeed [10]	1991	*Journal of the Pakistan Medical Association*	Case report	/	/	1	1	/	0:1 (female)	/	/	24	/	/	620	/	/	Single case of hypernatremia	/	Moderate (JBI critical appraisal tool)
Leslie et al. [11]	1995	*Journal of Paediatrics and Child Health*	Case–control	Inborn, GA ^a^ <28 wks or BW <1000 g	Not specified	102 infants available for matching	12 total (5 moderate SNHL ^c^ (40–70 dB), 5 moderate–severe (70–90 dB), 2 profound (>90 dB), 2 combined SNHL ^c^ and conductive HL ^c^)	24	1.40:1	7:5	14:10	/	26 ± 1.2	26 ± 1.1	/	982 ± 123	922 ± 159	Bilateral SNHL ^c^ >40 dB requiring hearing aids	Infants from same high-risk group without hearing loss matched for GA ^a^, sex and BW ^b^ (within 200 g)	High (NOS criteria)
Gawlowski et al. [12]	2006	*Journal of Paediatrics and Child Health*	Retrospective cohort	GA ^a^ <27 wks	Death within 5 DOL ^d^, transfers after 6 h, major congenital anomalies	66	46	20	1.54:1	30:16	10:10	25 (23–26)	24.5 (23–26)	26 (23–26)	710 (445–1030)	700 (535–1017)	770 (445–1030)	Hypernatremic infants	Normonatremic infants	Moderate (NIH quality assessment tool)
Sabir et al. [13]	2010	*Neonatology*	Case report	/	/	1	1	/	1:0 (male)	/	/	24 + 6	/	/	520	/	/	Single case of hypernatremia	/	High (JBI critical appraisal tool)
Lim et al. [5]	2011	*Journal of Perinatology*	Case–control	Inborn, BW ^b^ ≤1000 g, GA ^a^ ≤26 wks	Brain malformations, dysmorphic features, congenital anomalies, metabolic disorders, chromosomal disorders, confirmed CNS ^e^ infections in first 3 DOL ^d^	347 infants available for matching	36	36	1.77:1	28:8	18:18	Not reported	24.6 ± 1.0	24.8± 0.9	Not reported	764 ± 118.5	771.5 ± 125.9	Severe IVH ^f^ (grade III/IV)	No or grade I/II IVH ^f^, matched for GA ^a^ (±1 week) and BW ^b^ (±100 g). Matching failed on sex	High (NOS criteria)
Lee et al. [6]	2015	*Journal of Korean Medical Science*	Retrospective cohort	Inborn, preterm, BW ^b^ <1000 g	Congenital cardiac/renal anomalies, death within first 4 DOL ^d^, IVH ^f^ identified first 24 h of life	169	32	137	1.09:1	13:19	75:62	27 ± 2.6	25.4 ± 1.7	27.9 ± 2.5	771.8 ± 154.1	713.7 ± 158.7	785.4 ± 150.4	Severe IVH ^f^ (grade III/IV)	No or grade I/II IVH ^f^	High (NIH quality assessment tool)
Dalton et al. [4]	2015	*American Journal of Perinatology*	Retrospective cohort	Inborn, GA ^a^ <29 wks, BW ^b^ <1000 g	Death on DOL ^d^ 1, transfer after DOL ^d^ 3, no early CUS ^g^ insufficient sodium measurements (<every 12 h first 10 DOL)	216	126	90	1.06:1	69:57	42:48	Not reported	25.2 ± 0.1	25.9 ± 0.1	Not reported	736 ± 14	804 ± 14	Hypernatremia	No hypernatremia	High (NIH quality assessment tool)
Monnikendam et al. [3]	2019	*Journal of Perinatology*	Retrospective cohort	Inborn, BW ^b^ ≤1000 g, GA ^a^ 23–29 wks	Death or transfer within 7 DOL ^d^, no daily sodium data in first 7 DOL ^d^, congenital anomalies	12,428 in total, data of 12 199 presented	Group (−2 to 0): 96 Group (−1 to 0): 2223 Group (−1 to 1): 1814 Group (0 to 1): 5431 Group (0 to 2): 671	Group (0 to 0): 1964	0.93:1	Group (−2 to 0): 45:51 Group (−1 to 0): 988:1235 Group (−1 to 1): 892:922 Group (0 to 1): 2728:2703 Group (0 to 2): 385:286	Group (0 to 0): 828:1136	Not reported	Group (−2 to 0): 25.9 ± 1.7 Group (−1 to 0): 26.3 ± 1.6 Group (−1 to 1): 25.6 ± 1.6 Group (0 to −1): 25.6 ± 1.5 Group (0 to 2): 24.5 ± 1.3	Group (0 to 0): 26.4 ± 1.6	Not reported	Group (−2 to 0): 754 ± 154 Group (−1 to 0): 786 ± 140 Group (−1 to 1): 759 ± 146 Group (0 to 1): 755 ± 146 Group (0 to 2): 662 ± 139	Group (0 to 0): 788 ± 140	Various dysnatremic groups	Eunatremic group (class 0-0)	High (NIH quality assessment tool)
Späth et al. [14]	2022	*Acta Paediatrica*	Matched nested case–control	Live-born infants, GA ^a^ 22–26 + 6 wks, who survived first 24 h	No CUS ^g^, missing P-Na ^h^ data on day 2–3, major congenital or chromosomal anomalies	533 infants available for matching	70	70	1:1	35:35	35:35	25.3 ± 1/1	25.0 ± 1.0	25.0 ± 1.1	763 ± 169	741 ± 144	736 ± 140	Severe IVH ^f^ (grade III or PVHI ^i^)	No IVH ^f^, matched for hospital, sex, GA ^a^ (±1 week) and BW ^b^ (±170 g)	High (NOS criteria)
Gervais et al. [15]	2022	*Pediatric Research*	Retrospective cohort	≤28 wks GA ^a^, inborn or admitted within 48 h of life	Death within first 10 DOL ^d^	147 infants, neurodevelopmental outcome available for 114	3 groups of decreasing GA ^a^: <25 wks: N = 38 25–26 wks: N = 54 27–28 wks: N = 55	/	Total: 0.79:1. Per GA ^a^: <25 wks: 15:23 25–26 wks: 20:34 27–28 wks: 30:25	/	/	<25 wks: 24.3 ± 0.6 25–26 wks: 26.1 ± 0.6 27–28 wks: 28 ± 0.5	/	/	<25 wks: 682 ± 125 25–26 wks: 867 ± 160 27–28 wks: 1002 ± 233	/	/	Infants GA ^a^ ≤28 wks	/	High (NIH quality assessment tool)

Table legend: ^a^ Gestational age, ^b^ birth weight, ^c^ sensory neuronal hearing loss, ^d^ days of life, ^e^ central nervous system, ^f^ intraventricular hemorrhage, ^g^ cranial ultrasound, ^h^ plasma sodium, ^i^ periventricular hemorrhage infarction.

**Table 2:** Exposure (sodium-related data) of included studies.

Author	Exposure (Plasma Sodium)	Definition of Dysnatremia	Incidence (%)	Sodium Value(s) (mmol/L)	Day(s) of Life	Serum Sodium Trend
M. A. Saeed [10]	Extreme hypernatremia	Not defined	/(single case)	204	Between 60 and 72 h of life	S-Na ^a^ levels normalized by 96 h of life
Leslie et al. [11]	Hyponatremia, hypernatremia	Hyponatremia = P-Na ^b^ <125 mmol/L. Hypernatremia not defined.	Not reported	Not reported	Not specified	Not reported
Gawlowski et al. [12]	Hypernatremia	Hypernatremia = P-Na ^b^ >145 mmol/L at least twice.	69.7 (30.3% >150 mmol/L)	Not reported	DOL ^c^ 0–5	Hypernatremia most frequently between 24 and 72 h, peak at 48 h of age. Normal levels by DOL ^c^ 5–7.
Sabir et al. [13]	Severe hypernatremia	Hypernatremia = S-Na ^a^ >150 mmol/L.	/(single case)	199	DOL ^c^ 7	Serum sodium reduced within 72 h to 146 mmol/L
Lim et al. [5]	S-Na ^a^ fluctuations	Fluctuations in sodium = maximum − minimum S-Na ^a^ value (13 mmol/L = cut-off significant fluctuation). Hypernatremia not defined.	Not reported	Highest mean S-Na ^a^ value: 162.6 (cases), 148.8 (controls). Lowest mean S-Na ^a^ value: 145.3 (cases), 142.5 (controls).	DOL ^c^ 0–3	Not reported
Lee et al. [6]	Hypernatremia, hyponatremia, S-Na ^a^ fluctuations	Hypernatremia = S-Na ^a^ >150 mmol/L. Hyponatremia = S-Na ^a^ <130 mmol/L. Fluctuations in sodium = maximum − minimum S-Na ^a^ value.	Hypernatremia: 12.5% cases, 3.6% controls (total incidence = 5.3%). Hyponatremia: 3.2% cases, 2.2% controls, (total incidence = 2.3%).	Maximum mean S-Na ^a^ value: 144.7 ± 6.7 (cases), 142.2 ± 4.7 (controls). Minimum mean S-Na ^a^ value: 137.9 ± 5.0 (cases), 137.3 ± 4.2 (controls).	DOL ^c^ 0–4	Not reported
Dalton et al. [4]	Hypernatremia, rapid S-Na ^a^ fluctuations	Hypernatremia = S-Na ^a^ ≥150 mmol/L. Rapid rise/fall (≥10 and ≥15 mmol/L/day) of S-Na ^a^ in hypernatremic infants.	Hypernatremia 58%	Maximum median S-Na ^a^ level in hypernatremic group = 153 (range 150–181).	DOL ^c^ 0–10	Maximum S-Na ^a^ value on median postnatal age of 4 days
Monnikendam et al. [3]	Dysnatremia groups: severe hyponatremia to normal (class −2 to 0), moderate hyponatremia to normal (−1 to 0), moderate hyponatremia to moderate hypernatremia (−1 to 1), normal to moderate hypernatremia (0 to 1), normal to severe hypernatremia (0 to 2)	Degrees of dysnatremia (minimum and maximum S-Na ^a^ values): severe hyponatremia (<125 mEq/L, class −2), moderate hyponatremia (125–34 mEq/L, class −1), eunatremia (135–144 mEq/L, class 0), moderate hypernatremia (145–154 mEq/L, class 1), and severe hypernatremia (>154 mEq/L, class 2).	Hypernatremia: 65.4%. Hyponatremia: 35.1%. Hypo- and hypernatremia: 16.4%. Isolated moderate hypernatremia (group 0 to 1) most frequent (43.7%).	Mean S-Na ^a^ values remained within normal range, except among 23–24 wks. GA ^d^ infants: mean S-Na ^a^ 146 mmol/L (DOL ^c^ 2).	DOL ^c^ 0–7	S-Na ^a^ peak values around DOL ^c^ 2, return to baseline by DOL ^c^ 4
Gervais et al. [15]	Sodium and glucose fluctuations	Sodium and glucose fluctuations = maximum − minimum sodium/glucose value. P-Na ^b^ corrected for glucose using Katz formula (Na(corr) = Na + 0.3 × (Glucose -5)).	Not reported	Not reported	DOL 0–10	Increase first 2 DOL ^c^, followed by rapid decrease. Decrease larger with lower GA ^c^. Glucose-corr sodium levels variability increaded with decreasing GA ^c^.
Späth et al. [14]	Hypernatremia, P-Na ^b^ fluctuations	3 levels of hypernatremia defined: P-Na ^b^ >145 mmol/L, P-Na ^b^ >150 mmol/L and P-Na ^b^ >155 mmol/L. Fluctuation in P-Na ^b^ = difference peak-nadir values birth to DOL ^c^ 3.	Total incidence of hypernatremia = 75.7%. Per supgroup: P-Na ^b^ >145 mmol/L: 40% (28 cases), 58.6% (41 controls). P-Na ^b^ >150 mmol/L: 17.1% (12 cases), 24.3% (17 controls). P-Na ^b^ >155 mmol/L: 7.1% (5 cases, 4.3% (3 controls).	Highest mean P-Na ^b^ concentration: 144.9 ± 5.8 mmol/L (cases), 146.6 ± 5.25 mmol/L (controls).	DOL ^c^ 0–3	Not reported

Table legend: ^a^ Serum sodium, ^b^ plasma Sodium, ^c^ day of life, ^d^ gestational age.

**Table 3:** Neurodevelopmental outcomes of included studies.

Author	Neurodevelopmental Outcome (Measurement)	Timing of Outcome	Outcome Measure	Statistics	Key Findings	Power
M. A. Saeed [10]	Mental and physical development (Denver scale)	6 months, 1 year and 2 years CA ^a^	Normal neurodevelopment	/	A case of severe hypernatremia with supsequent normal neurodevelopment	/
Leslie et al. [11]	Significant SNHL ^b^ (visual reinforcement, orientation audiometry or brainstem auditory evoked responses)	8–10 months of CA ^a^	Categorized variables significantly associated with hearing loss: P-Na ^c^ <125 mmol/L, OR = 5.6 (*p* = 0.03)	Univariate and multivariate testing, categorized variables	Hyponatremia, but not hypernatremia, is a significant risk factor for SNHL ^b^	Power of study low (number of cases and controls small, number of variables examined extensive)
Gawlowski et al. [12]	Severe IVH ^d^ grade III/IV (CUS ^e^, Papile classification)	Not specifically mentioned, infants studied DOL ^f^ 0–5	Incidence of severe IVH ^d^: 8 cases (17.4%) vs. 0 controls, (*p* = 0.66)	Univariate testing	No significant difference in development of severe IVH ^d^ between hypernatremic and normonatremic infants	Not mentioned, small sample size
Sabir et al. [13]	Brain bleed/edema after event (CUS ^e^), structural deficits (MRI), neurodevelopmental evaluation on mental and motor scales (Bayley Scales of Infant Development-II)	MRI at 4 months CA ^a^, Bayley Scales at 6 months CA ^a^	Normal CUS ^e^, normal MRI, normal neurodevelopmental evaluation by Bayley Scales	/	A case of severe hypernatremia with supsequent normal neurodevelopment	/
Lim et al. [5]	Severe IVH ^d^: grade III/IV (routine CUS ^e^, Papile classification), PVL ^g^ (CUS ^e^), PHH ^h^ (CUS ^e^ or CT)	DOL ^f^ 0–3	Incidence of severe IVH ^d^ = 10.7% (37/347). Univariate analysis IVH ^d^ group: higher maximal S-Na ^h^ level (162.6 vs. 148.8 mmol/L; *p* < 0.001), more prominent S-Na ^h^ fluctuation (17.3 vs. 6.2 mmol/L; *p* < 0.001), more changes in S-Na ^h^ >13 mmol/L (*p* < 0.001) Multivariate regression: only fluctuations S-Na ^h^ >13 mmol/L increased risk of IVH ^d^ (OR 12.4; 95% CI 1.8–82.7)	Significant values associated with IVH ^d^ by univariate analysis used in stepwise logistic regression model	Higher S-Na ^h^ levels and larger S-Nah fluctuations are risk factors for severe IVH ^d^ on univariate analysis only. After multivariate analysis, fluctuations of S-Na ^h^ levels >13 mmol/L were strongly associated with severe IVH ^d^.	Not mentioned, small number of cases and controls
Lee et al. [6]	Severe IVH ^d^ grade III/IV (routine CUS ^e^, Papile classification)	DOL ^f^ 0–10	Incidence of severe IVH ^d^: 18.9% (n = 32). No difference in incidence of hypernatremia (*p* = 0.067), hyponatremia (*p* = 0.572), maximum (*p* = 0.058)/minimum (*p* = 0.496) S-Na ^h^ level and degree of sodium fluctuation (*p* = 0.018).	Univariate and multivariate analysis	No significant correlation between S-Na ^h^ concentration in first 4 DOL ^f^ and severe IVH ^d^	Not mentioned, small sample size
Dalton et al. [4]	Any IVH ^d^, severe IVH ^d^ = grade III-IV, cystic PVL ^g^ (CUS ^e^, Papile classification), composite outcome of severe IVH ^d^ or death by day 10	DOL ^f^ 0–10, median postnatal age any IVH ^d^ = 5 days, severe IVH ^d^ = 4 days	Hypernatremic infants vs. non-hypernatremic group: Incidence of: Any IVH ^d^: 63% vs. 46% (*p* = 0.01) Severe IVH ^d^: 29% vs. 19% (*p* = 0.11) Cystic PVL ^g^: 13% vs. 8% (*p* = 0.27)	Variables significantly associated with any IVH ^d^ in univariate analysis used in stepwise forward logistic regression model	Hypernatremic infants show significantly higher incidence of any IVH ^d^, but not of severe IVH or cystic PVL ^g^	Not mentioned, small sample size
Monnikendam et al. [3]	Severe IVH ^d^: grade III-IV (CUS ^e^, Papile classification)	After DOL ^f^ 7	Incidence of severe IVH ^d^, compared to group 0 to 0 (8.0%): Group −2 to 0: 28.9% (*p* < 0.001) Group −1 to 0: 12.5% (*p* < 0.001) Group −1 to 1: 17.7% (*p* < 0.05) Group 0 to 1: 11.6% (*p* < 0.05) Group 0 to 2: 27.4% (*p* < 0.001)	Univariate analysis	Dysnatremic groups had significantly higher rates of severe IVH ^d^ compared to normal-level-sodium group	Not mentioned, large sample size
Gervais et al. [15]	Neurodevelopmental outcomes (examination by pediatrician, Bayley Scales of Infant and Toddler Development-III, audiology and ophthalmology reports). Impairment defined as cerebral palsy, Bayley score <85, blindness or hearing loss requiring amplification.	18–21 months CA ^a^	Association (using coefficients B (95% CI)) of glucose-corr P-Na ^c^ fluctuations and death or neurodevelopmental impairment at 18 months CA ^a^: Adjustment for GA ^i^: 3.19 (*p* = 0.036), Adjustment for all covariates (=GA ^i^, SNAP score, NSAID use): 2.03 (*p* = 0.087)	Unadjusted comparison between 3 groups of decreasing GAi. Linear models for variables to study association.	Glucose-corr sodium fluctuations during first 10 days of life significantly associated with death or neurodevelopmental impairment at 18 months CA ^a^, after adjustment for GA ^i^, but not after adjustment for all covariates	Not mentioned, small sample size
Späth et al. [14]	Severe IVH ^d^: grade III or PVHI^j^ (routine CUS ^e^, papille classification)	Exact time points of IVH ^d^ detection not mentioned, but diagnosed by routine early CUS ^e^	Incidence of severe IVH ^d^ = 13.5% (72/533). Comparison between cases/controls (OR): Highest P-Na ^c^ concentration: 1.001 (*p* = 0.990) P-Na ^c^ fluctuations: 1.002 (*p* = 0.649) P-Na ^c^ >145 mmol/L: 1.028 (*p* = 0.958) P-Na ^c^ >150 mmol/L: 0.945 (*p* = 0.924) P-Na ^c^ >155 mmol/L: 5.374 (*p* = 0.263).	Multivariable logistic regression model	Peak P-Na ^c^ levels; hypernatremia and large fluctuations in P-Na ^c^ were not associated with increased risk of severe IVH ^d^	Post hoc power analyses (70 cases, 1 matched control for each case): power to detect a difference between cases and controls = 82% for continuous explanatory variables using a two-tailed test, an effect size of 0.35 and a significance level of 5%

Table legend: ^a^ Corrected age, ^b^ sensory neuronal hearing loss, ^c^ plasma sodium, ^d^ intraventricular hemorrhage, ^e^ cranial ultrasound, ^f^ DOL = days of life, ^h^ serum sodium, ^g^ PVL = periventricular leukomalacia, ^h^ post-hemorrhagic hydrocephalus, ^i^ gestational age, ^j^ periventricular hemorrhagic infarction.

## Data Availability

No new data were created or analyzed in this study. Data sharing is not applicable to this article.

## Supplementary Materials

The following supporting information can be downloaded at: https://www.mdpi.com/article/10.3390/children13020287/s1, Table S1: Search strategy used in PubMed, Embase, Web of Science Core Collection, CINAHL, and Scopus; File S1: Risk-Bias analysis and quality assessment.

## References

[1] Rogers E.E., Hintz S.R. (2016). Early neurodevelopmental outcomes of extremely preterm infants. Semin. Perinatol..

[2] Zayegh A.M., Doyle L.W., Boland R.A., Mainzer R., Spittle A.J., Roberts G., Hickey L.M., Anderson P.J., Cheong J.L. (2022). Trends in survival, perinatal morbidities and two-year neurodevelopmental outcomes in extremely low-birthweight infants over four decades. Paediatr. Perinat. Epidemiol..

[3] Monnikendam C.S., Mu T.S., Aden J.K., Lefkowitz W., Carr N.R., Aune C.N., Ahmad K.A. (2019). Dysnatremia in extremely low birth weight infants is associated with multiple adverse outcomes. J. Perinatol..

[4] Dalton J., Dechert R.E., Sarkar S. (2015). Assessment of association between rapid fluctuations in serum sodium and intraventricular hemorrhage in hypernatremic preterm infants. Am. J. Perinatol..

[5] Lim W.H., Lien R., Chiang M.C., Fu R.H., Lin J.J., Chu S.M., Hsu J.F., Yang P.H. (2011). Hypernatremia and grade III/IV intraventricular hemorrhage among extremely low birth weight infants. J. Perinatol..

[6] Lee H.J., Lee B.S., Do H.J., Oh S.H., Choi Y.S., Chung S.H., Kim E.A., Kim K.S. (2015). Early sodium and fluid intake and severe intraventricular hemorrhage in extremely low birth weight infants. J. Korean Med. Sci..

[7] Marcialis M.A., Dessi A., Pintus M.C., Marinelli V., Fanos V. (2012). Hyponatremia and hypernatremia in the newborn: In medio stat virtus. Front. Biosci. Elite.

[8] Covidence Systematic Review Management Software. https://www.covidence.org.

[9] Page M.J., McKenzie J.E., Bossuyt P.M., Boutron I., Hoffmann T.C., Mulrow C.D., Shamseer L., Tetzlaff J.M., Akl E.A., Brennan S.E. (2021). The PRISMA 2020 statement: An updated guideline for reporting systematic reviews. BMJ.

[10] Saeed M.A. (1991). Severe hypernatraemia in a very preterm infant 2006. J. Pak. Med. Assoc..

[11] Leslie G., Kalaw M., Bowen J., Arnold J. (1995). Risk factors for sensorineural hearing loss in extremely premature infants. J. Paediatr. Child. Health.

[12] Gawlowski Z., Aladangady N., Coen P.G. (2006). Hypernatraemia in preterm infants born at less than 27 weeks gestation. J. Paediatr. Child. Health.

[13] Sabir H., Stannigel H., Mayatepek E., Hoehn T. (2010). Severe hypernatremia in an extremely low birth weight infant with subsequent normal neurological development. Neonatology.

[14] Späth C., Stoltz Sjöström E., Ågren J., Ahlsson F., Domellöf M. (2022). Sodium supply from administered blood products was associated with severe intraventricular haemorrhage in extremely preterm infants. Acta Paediatr..

[15] Gervais A.S., Luu T.M., Viennet A., Milette A.A., Vallée J., Cloutier A., Lefebvre F., Nuyt A.M., Flahault A. (2022). Neurodevelopmental consequences of early plasma sodium changes in very preterm infants. Pediatr. Res..

[16] Durrani N.U.R., Imam A.A., Soni N. (2022). Hypernatremia in Newborns: A Practical Approach to Management. Biomed. Hub..

[17] Zamir I., Stoltz Sjöström E., Ahlsson F., Hansen-Pupp I., Serenius F., Domellöf M. (2021). Neonatal hyperglycaemia is associated with worse neurodevelopmental outcomes in extremely preterm infants. Arch. Dis. Child. Fetal Neonatal Ed..

[18] Becker K., Becker H., Riedl-Seifert T., Waitz M., Jenke A. (2024). Excessive sodium supplementation but not fluid load is correlated with overall morbidity in extremely low birth weight infants. JPGN Rep..

[19] Sas Svan Pace M., Salaets T., Laenen A., Raaijmakers A., Allegaert K. (2025). Sodium Patterns and Their Variables in a Cohort of ELBW Infants in the First 10 Days of Life. Children.

[20] Pace M., van Sas S., Salaets T., Laenen A., Raaijmakers A., Allegaert K. (2025). Hypo- and Hypernatremia in Extremely Low Birth Weight Infants in the First 10 Days of Life: A Review. Children.

[21] Hong J., Rha D.W. (2023). The Long-Term Outcome and Rehabilitative Approach of Intraventricular Hemorrhage at Preterm Birth. J. Korean Neurosurg. Soc..

[22] Lorenz J.M., Kleinman L.I., Kotagal U.R., Reller M.D. (1982). Water balance in very low-birth-weight infants: Relationship to water and sodium intake and effect on outcome. J. Pediatr..

[23] Brown D.R., Watchko J.F., Sabo D. (1991). Neonatal sensorineural hearing loss associated with furosemide: A case-control study. Dev. Med. Child. Neurol..

[24] Allegaert K., Hildebrand H., Singh K., Turner M.A. (2023). The publication quality of laboratory values in clinical studies in neonates. Pediatr. Res..

[25] Bermick J., Dechert R.E., Sarkar S. (2016). Does hyperglycemia in hypernatremic preterm infants increase the risk of intraventricular hemorrhage?. J. Perinatol..

[26] van der Lugt N.M., Smits-Wintjens V.E.H.J., van Zwieten P.H.T., Walther F.J. (2010). Short and long term outcome of neonatal hyperglycemia in very preterm infants: A retrospective follow-up study. BMC Pediatr..

