# Supplementary material for: Sodium Values During the First 10 Postnatal Days in Extremely-Low-Birth-Weight Infants and Long-Term Neurocognitive Outcomes: A Systematic Review
Source: Children (Basel). 2026 Feb 19;13(2):287. doi: 10.3390/children13020287 (PMC12939160; doi:10.3390/children13020287)
Supplement: Supplementary file 1 [file children-13-00287-s001.zip › File S1.pdf]

File S1: Risk-Bias analysis and quality assessment

Author	Level of quality
M. A. Saeed [10]	Moderate
Leslie et al.[11]	High
Gawlowski et al.[12]	Moderate
Sabir et al.[13]	High
Lim et al.[5]	High
Lee et al.[6]	High
Dalton et al.[4]	High
Monnikendam et al.[3]	High
Gervais et al.[15]	High
Späth et al.[14]	High

Author	Tool	Level of quality
M.A Saeed [10]	JBI Checklist Evaluation	Moderate
Leslie et al.[11]	Newcastle-Ottawa Scale Criteria (NOS)	High
Gawlowski et al.[12]	NIH Quality Assessment Tool	Moderate
Sabir et al.[13]	JBI Checklist Evaluation	High
Lim et al.[5]	Newcastle-Ottawa Scale Criteria (NOS)	High
Lee et al.[6]	NIH Quality Assessment Tool	High
Dalton et al.[4]	NIH Quality Assessment Tool	High
Monnikendam et al.[3]	NIH Quality Assessment Tool	High
Gervais et al.[15]	NIH Quality Assessment Tool	High
Späth et al.[14]	Newcastle-Ottawa Scale Criteria (NOS)	High

How this was obtained:

Retrospective cohort studies (5):

Tool = NIH quality assessment tool
Study quality rated as low (0-4 out of 14 questions), moderate (5-10 out of 14 questions), or high (11-14 out of 14 questions)

1. **"Hypernatraemia in preterm infants born at less than 27 weeks gestation"**
   By Gawlowski et al.[12]

Criteria	Yes	No	Uncertain/not applicable	Notes
**Was the research question or objective in this paper clearly stated?**	X			Primary and secondary objectives are clearly stated in the introduction.
**Was the study population clearly specified and defined?**	X			Study population was clearly defined in methods' section.
**Was the participation rate of eligible persons at least 50%?**	X			83 infants admitted, 66 were included = participation rate 79.5%

**Were all the subjects selected or recruited from the same or similar populations (including the same time period)? Were inclusion and exclusion criteria for being in the study prespecified and applied uniformly to all participants?**	X			All participants were selected from the same neonatal unit during the same time period. Inclusion criteria and exclusion criteria were uniformly applied.
**Was a sample size justification, power description, or variance and effect estimates provided?**		X		The study did not provide a sample size justification or power description. No explanation of how the sample size was determined. This might be a limitation in evaluating whether the study had sufficient power to detect associations.
**For the analyses in this paper, were the exposure(s) of interest measured prior to the outcome(s) being measured?**	X			Hypernatremia was measured from birth until minimal day 5 of life, before complications or outcomes were measured.
**Was the timeframe sufficient so that one could reasonably expect to see an association between exposure and outcome if it existed?**	X			The study followed the infants for at least 5 days, which is a reasonable timeframe as complications like IVH typically manifest within the first few days in this population.
**For exposures that can vary in amount or level, did the study examine different levels of the exposure as related to the outcome (e.g., categories of exposure, or exposure measured as continuous variable)?**		X		The study did not examine varying levels of hypernatraemia but classified infants into hypernatraemic (>145 mmol/L) and non-hypernatraemic groups.
**Were the exposure measures (independent variables) clearly defined, valid, reliable, and implemented consistently across all study participants?**	X			The exposure measure was cleary defined, and is a valid and reliable measure for hypernatremia. It was consistently measured across all participants, standard biochemical tests were used and are reliable and valid.
**Was the exposure(s) assessed more than once over time?**	X			Checks of plasma sodium every 12h in the first 2 days, after this every 24h, until at least day 5.
**Were the outcome measures (dependent variables) clearly defined, valid, reliable, and implemented consistently across all study participants?**	X			Outcome measures were defined (eg complications: IVH, CLD, PDA, NEC). These outcomes were measures consistently. Assessment was based on standard clinical criteria and protocols.
**Were the outcome assessors blinded to the exposure status of participants?**		X		No mention of blinding of outcome assessors.

**Was loss to follow-up after baseline 20% or less?**	X			For 8 infants medical records were incomplete for fluid and sodium intake. (=12%); this was not the case for other outcomes. There was no loss to follow up mentioned.
**Were key potential confounding variables measured and adjusted statistically for their impact on the relationship between exposure(s) and outcome(s)?**		X		No statistical adjustment for potential confounders. Only univariate comparison was made.

Quality rating: 10 yes, 4 no = **moderate quality.**

- **Strengths**: clear defined research question and study population, well-established measure for exposure and outcomes. Timeframe is appropriate, exposure assessed multiple times.
- **Weaknesses**: No justification of sample size, no statistical adjustment for confounders, lack of blinding introducing possible bias**.**

2. **"Early Sodium and Fluid Intake and Severe Intraventricular Hemorrhage in Extremely Low Birth Weight Infants"** by Lee et al.[6]

**Criteria**	**Yes**	**No**	**Uncertain/not applicable**	**Notes**
**Was the research question or objective in this paper clearly stated?**	x			Research question was clearly stated in the introduction.
**Was the study population clearly specified and defined?**	x			Clear defined study population in methods' section.
**Was the participation rate of eligible persons at least 50%?**	x			210 infants admitted, 169 included = more than 80% participation rate.
**Were all the subjects selected or recruited from the same or similar populations (including the same time period)? Were inclusion and exclusion criteria for being in the study prespecified and applied uniformly to all participants?**	x			Subjects selected from same NICU, during same period. Inclusion and exclusion criteria uniformly applied to participants.
**Was a sample size justification, power description, or variance and effect estimates provided?**		x		No sample size justification or statistical power calculation, no specification of how number of participants needed was determined.
**For the analyses in this paper, were the exposure(s) of interest measured prior to the outcome(s) being measured?**	x			Serum sodium, sodium and fluid intake (exposure) was measured from birth until day 4, before outcome was determined.
**Was the timeframe sufficient so that one could reasonably expect to see an association**	x			Timeframe of sodium monitoring was first 4 days of life, with follow-up until day 10 for IVH assessment. This is sufficient.

**between exposure and outcome if it existed?**				
**For exposures that can vary in amount or level, did the study examine different levels of the exposure as related to the outcome (e.g., categories of exposure, or exposure measured as continuous variable)?**	x			For the exposure measures fluid and sodium intake, varying levels of intake were examined.
**Were the exposure measures (independent variables) clearly defined, valid, reliable, and implemented consistently across all study participants?**	x			Exposure measures (fluid and sodium intake, hypernatremia and hyponatremia) cleary defined, measured reliably and implemented consistently among all participants.
**Was the exposure(s) assessed more than once over time?**	x			Exposure was assessed daily for first 4 days of life.
**Were the outcome measures (dependent variables) clearly defined, valid, reliable, and implemented consistently across all study participants?**	x			IVH severity using Papile classification system by routine cranial ultrasound. This is a valid and reliable way to measure outcome. Implemented consistently.
**Were the outcome assessors blinded to the exposure status of participants?**		x		No blinding mentioned.
**Was loss to follow-up after baseline 20% or less?**	x			No loss to follow-up reported. All 169 infants monitored consistently.
**Were key potential confounding variables measured and adjusted statistically for their impact on the relationship between exposure(s) and outcome(s)?**	x			The study used multivariate analysis to adjust for potential confounders, reducing confounding bias.

Quality rating: 12 yes, 2 no = **high quality**

- **Strengths:** Clear research question, well defined study population. Clear definitions of exposure and outcome measures, and reliable methods used to measure these. Adjustment for important confounders, making analysis more robust.
- **Weaknesses:** No sample size justification or power analysis. No blinding, introducing possible bias.

3. **"Assessment of Association between Rapid Fluctuations in Serum Sodium and Intraventricular Hemorrhage in Hypernatremic Preterm Infants"** by Dalton et al.[4]

**Criteria**	**Yes**	**No**	**Uncertain/not applicable**	**Notes**
**Was the research question or objective in this paper clearly stated?**	x			Objective clearly defined in the introduction.

**Was the study population clearly specified and defined?**	x			Study population clearly defined in methods' section.
**Was the participation rate of eligible persons at least 50%?**	x			304 infants admitted, 216 included = participation rate of 71%
**Were all the subjects selected or recruited from the same or similar populations (including the same time period)? Were inclusion and exclusion criteria for being in the study prespecified and applied uniformly to all participants?**	x			Subjects selected from same NICU, during same period. Inclusion and exclusion criteria uniformly applied to participants.
**Was a sample size justification, power description, or variance and effect estimates provided?**		x		No sample size justification or power analysis. No specification of how number of participants needed was determined.
**For the analyses in this paper, were the exposure(s) of interest measured prior to the outcome(s) being measured?**	x			Sodium levels (exposure) were monitored during first 10 days of life every 12h, prior to outcome (IVH, death during first 10 days).
**Was the timeframe sufficient so that one could reasonably expect to see an association between exposure and outcome if it existed?**	x			Participants were followed for first 10 days of life, which was sufficient to monitor outcomes.
**For exposures that can vary in amount or level, did the study examine different levels of the exposure as related to the outcome (e.g., categories of**	x			Different levels of fluctuation in serum sodium were analyzed and related to the outcomes.

**exposure, or exposure measured as continuous variable)?**				
**Were the exposure measures (independent variables) clearly defined, valid, reliable, and implemented consistently across all study participants?**	x			Sodium fluctuations were clearly defined and measured consistently across all participants, using valid and reliable practiced for measurement.
**Was the exposure(s) assessed more than once over time?**	x			At least every 12h, allowing for repeated measurements of exposure.
**Were the outcome measures (dependent variables) clearly defined, valid, reliable, and implemented consistently across all study participants?**	x			Outcome measures (IVH, death) were clearly defined. A reliable and validated method for detection of IVH was used, namely cranial ultrasound and Papile classification.
**Were the outcome assessors blinded to the exposure status of participants?**		x		Blinding was not mentioned.
**Was loss to follow-up after baseline 20% or less?**	x			No loss to follow-up was mentioned. All 216 included infants were analyzed.
**Were key potential confounding variables measured and adjusted statistically for their impact on the relationship between exposure(s) and outcome(s)?**	x			Adjustment for potential confounders was done through logistic regression analysis.

Quality rating: 12 yes, 2 no = **high quality**

- **Strengths:** Clear research question**,** well defined study population. Clear definitions of exposure and outcome measures, and reliable methods used to measure these. Adjustment for important confounders, making analysis more robust.
- **Weaknesses:** No sample size justification or power analysis. No blinding, introducing possible bias.

4. **"Dysnatremia in extremely low birth weight infants is associated with multiple adverse outcomes"** by Monnikendam et al.[3]

Criteria	Yes	No	Uncertain/not applicable	Notes
**Was the research question or objective in this paper clearly stated?**	x			Objective clearly stated in introduction.
**Was the study population clearly specified and defined?**	x			Study population clearly defined in methods' section.
**Was the participation rate of eligible persons at least 50%?**		x		Initial cohort of 26.871, inclusion of 12.428 infants = participation rate of 46%
**Were all the subjects selected or recruited from the same or similar populations (including the same time period)? Were inclusion and exclusion criteria for being in the study prespecified and applied uniformly to all participants?**	x			Subjects selected from same NICU, during same period. Inclusion and exclusion criteria uniformly applied to participants.
**Was a sample size justification, power description, or variance and effect estimates provided?**	x			No sample size justification or power analysis. No specification of how number of participants needed was determined.
**For the analyses in this paper, were the exposure(s) of interest measured prior to the outcome(s) being measured?**		x		Serum sodium levels (exposures) were measured within first 7 days of life, before outcomes (mortality, IVH,…) were measured.
**Was the timeframe sufficient so that one could reasonably expect to see an association between exposure and outcome if it existed?**	x			Study monitored serum sodium levels during first week of life, this is sufficient.
**For exposures that can vary in amount or level, did the study examine different levels of the exposure as related to the outcome (e.g., categories of exposure, or exposure measured as continuous variable)?**	x			Multiple levels of serum sodium were examined. Infants were categorized into different groups based on maximum and minimum sodium levels.
**Were the exposure measures (independent variables) clearly defined, valid, reliable, and implemented**	x			Serum sodium measurements were clearly defined and consistently implemented in all participants.

**consistently across all study participants?**				
**Was the exposure(s) assessed more than once over time?**	x			Serum sodium levels were assessed daily during first week of life, allowing multiple assessments of exposure over time.
**Were the outcome measures (dependent variables) clearly defined, valid, reliable, and implemented consistently across all study participants?**	x			Outcome measures, including IVH, ROP, PDA, BPD and NEC were defied bij using standard clinical criteria. Mortality was assessed before discharge. IVH classified by Papile classification, using cranial ultrasound. There was consistency across participants.
**Were the outcome assessors blinded to the exposure status of participants?**		x		Blinding not mentioned.
**Was loss to follow-up after baseline 20% or less?**	x			No significant loss to follow-up was reported.
**Were key potential confounding variables measured and adjusted statistically for their impact on the relationship between exposure(s) and outcome(s)?**	x			Adjustment for potential confounders was done by using multiple regression analysis.

Quality rating: 11 yes, 3 no = **high quality**

- **Strengths:** There is adjustment for confounders and the study uses a robust, multi-center database, making findings more generalizable. Further, there is a clear research question**,** and well defined study population. Clear definitions of exposure and outcome measures, and reliable methods used to measure these.
- **Weaknesses:** No sample size justification or power analysis. No blinding, introducing possible bias. Participation rate of 50%.

5. **"Neurodevelopmental consequences of early plasma sodium changes in very preterm infants"** by Gervais et al.[15]

**Criteria**	**Yes**	**No**	**Uncertain/not applicable**	**Notes**
**Was the research question or objective in this paper clearly stated?**	x			Study's objective clearly defined.
**Was the study population clearly specified and defined?**	x			Study population clearly defined.
**Was the participation rate of eligible persons at least 50%?**	x			Of 169 neonates born, 147 were included = participation rate of 87%
**Were all the subjects selected or recruited from the same or similar populations**	x			Subjects selected from same NICU, during same period. Inclusion and exclusion criteria uniformly applied to participants.

**(including the same time period)? Were inclusion and exclusion criteria for being in the study prespecified and applied uniformly to all participants?**				
**Was a sample size justification, power description, or variance and effect estimates provided?**		x		No sample size justification or power analysis. No specification of how number of participants needed was determined.
**For the analyses in this paper, were the exposure(s) of interest measured prior to the outcome(s) being measured?**	x			Plasma sodium and glucose fluctuations (exposure) were measured during first 10 days of life, this was well before outcome of death or neurodevelopment at 18 months corrected ages.
**Was the timeframe sufficient so that one could reasonably expect to see an association between exposure and outcome if it existed?**	x			Plasma and glucose levels from day 1 to 30 were examined, they found that variability was maximal in first 10 days of life. Thus, they specifically studied determinants and consequences of glucose-corrected sodium changes in first 10 days of life. The timeframe was sufficient.
**For exposures that can vary in amount or level, did the study examine different levels of the exposure as related to the outcome (e.g., categories of exposure, or exposure measured as continuous variable)?**	x			Varying levels of glucose-corrected plasma sodium were examined and their relationship with neurodevelopmental outcomes at 18 months corrected age were assessed.
**Were the exposure measures (independent variables) clearly defined, valid, reliable, and implemented consistently**	x			Plasma sodium and glucose measurements were clearly defined, and the Katz formula was used to correct sodium levels for glucose. The data were collected reliably from the biochemistry department.

**across all study participants?**				
**Was the exposure(s) assessed more than once over time?**	x			Plasma sodium and glucose levels were assessed daily from day 1 to 10, allowing multiple assessments of exposure over time.
**Were the outcome measures (dependent variables) clearly defined, valid, reliable, and implemented consistently across all study participants?**	x			Neurodevelopmental outcomes were clearly defined using a composite outcome of death or neurodevelopmental impairment, including cerebral palsy, hearing loss requiring amplification, blindness, or Bayley scores <85 (= standardized tool).
**Were the outcome assessors blinded to the exposure status of participants?**		x		No blinding mentioned
**Was loss to follow-up after baseline 20% or less?**	x			Due to death between day 11 and 18 months, and due to missing data due to COVID, neurodevelopmental outcomes for 114 infants (=85%) at 18 months was available. Maily missing data due to COVID19.
**Were key potential confounding variables measured and adjusted statistically for their impact on the relationship between exposure(s) and outcome(s)?**	x			The study adjusted for important confounders, including gestational age and SNAP severity score. This strengthens the validity of the results.

Quality rating: 12 yes, 2 no = **high quality**

- **Strengths:** Clear research question**,** well defined study population. Clear definitions of exposure and outcome measures, and reliable methods used to measure these. Adjustment for important confounders, making analysis more robust. Additionally, the use of glucose-corrected sodium provides a more accurate reflection of plasma osmolality, which is critical in this population.
- **Weaknesses:** No sample size justification or power analysis. No blinding, introducing possible bias. Some missing data could have affected the representativeness of the results.

Non-randomized, retrospective case-control studies (3):

Tool = Newcastle-Ottawa Scale criteria (NOS)
Quality assessment: counts stars: low (0-3 stars), moderate (4-6) and high (7-9).

1. **"Risk factors for sensorineural hearing loss in extremely premature infants"** by Leslie et al.[11]

Selection: 4 stars rating

	**Is case definition adequate?**	**Representativeness of cases**	**Selection of controls**	**Definition of controls**

Possible answers<br><br>(bold = answer for study)	**a) yes, with independent validation: ***<br>b) yes, eg record linkage or based on self reports<br>c) no description	**a) consecutive or obviously representative series of cases: ***<br>b) potential for selection biases or not stated	**a) community controls: ***<br>b) hospital controls<br>c) no description	a) no history of disease (endpoint): *<br>b) no description of source
**Applied to study**	Case definition for SNHL was adequately described, as infants with bilateral SNHL >40dB requiring hearing aids were identified and validated through formal audiological testing at a recognized hearing center.	All surviving infants with a gestational age (GA) <28 weeks or birthweight (BW) <1,000g admitted to the neonatal unit during the study period were included, ensuring a representative sample of cases of ELBW infants with SNHL.	Controls were matched based on GA, BW, and sex, and were from the same high-risk group of survivors.	Controls had no sensorineural or conductive hearing loss, which was explicitly stated

Comparability: 2 stars rating

	**Comparability of cases and controls on the basis of the design or analysis**
**Possible answers (bold = answer for study)**	**a) study controls for ### (Select the most important factor.) : ***<br>**b) study controls for any additional factor: * (This criteria could be modified to indicate specific control for a second important factor.)**
**Applied to study**	Study controls for age, BW and sex, therefor controlling for major potential confounders.

Exposure: 3 stars rating

	**Ascertainment of exposure**	**Same method of ascertainment for cases and controls?**	**Non-Response rate**
**Possible answers**<br><br>**(bold = answer for study)**	**a) secure record: ***<br>b) structured interview where blind to case/control status: *<br>c) interview not blinded to case/control status<br>d) written self report or medical record only<br>e) no description	**a) yes: ***<br>b) no	**a) same rate for both groups: ***<br>b) non respondents described<br>c) rate different and no designation
**Applied to study**	Review of medical records	Cases and controls were assessed for exposure using same procedures and medical records	No significant mention of non-respondents

Quality rating: 9/9 stars = **high quality**.
Good method in terms of case and control selection, exposure ascertainment and matching of variables. Low risk of bias based on NOS criteria.

Limitation: retrospective data from medical records, potential for information bias.

2. **"Hypernatremia and grade III/IV intraventricular hemorrhage among extremely low birth weight infants"** by Lim et al. [5]

Selection: 4 stars rating

	**Is case definition adequate?**	**Representativeness of cases**	**Selection of controls**	**Definition of controls**
**Possible answers**<br><br>**(bold = answer for study)**	**a) yes, with independent validation: ***<br>b) yes, eg record linkage or based on self reports<br>c) no description	**a) consecutive or obviously representative series of cases: ***<br>b) potential for selection biases or not stated	**a) community controls: ***<br>b) hospital controls<br>c) no description	**a) no history of disease (endpoint): ***<br>b) no description of source
**Applied to study**	Case definition was grade III/IV IVH, this was diagnosed using routine cranial ultrasound and Papile classification, a standard and validated method.	The study included all infants with a birth weight ≤1000 g and gestational age ≤26 weeks admitted to the NICU, ensuring a representative sample of cases of ELBW infants with severe IVH	Control group = infants without IVH, matched for GA and BW. Selected from same cohort, ensuring similarity of population.	It was explicitly stated that controls had no IVH, measured by cranial US.

Comparability: 2 stars rating

	**Comparability of cases and controls on the basis of the design or analysis**
**Possible answers (bold = answer for study)**	**a) study controls for ### (Select the most important factor.) : ***<br>**b) study controls for any additional factor: * (This criteria could be modified to indicate specific control for a second important factor.)**
**Applied to study**	Study controls for age, BW, and sex. Additionally, the analysis adjusted for potential confounders like mode of delivery and sodium fluctuations using multivariate logistic regression.

Exposure: 3 stars rating

	**Ascertainment of exposure**	**Same method of ascertainment for cases and controls?**	**Non-Response rate**
**Possible answers**<br><br>**(bold = answer for study)**	**a) secure record: ***<br>b) structured interview where blind to case/control status: *<br>c) interview not blinded to case/control status<br>d) written self report or medical record only<br>e) no description	**a) yes: ***<br>b) no	**a) same rate for both groups: ***<br>b) non respondents described<br>c) rate different and no designation
**Applied to study**	Medical records	Serum sodium levels and other relevant variables were monitored and collected by same protocol	No significant mention of non-respondents

Quality rating: 9/9 stars = **high quality**.
Good method in terms of case and control selection, exposure ascertainment and matching of variables. Low risk of bias based on NOS criteria.

Limitation: retrospective data from medical records, potential for information bias.

3. **"Sodium supply from administered blood products was associated with severe intraventricular haemorrhage in extremely preterm infants"** by Späth et al.[14]

Selection: 4 stars rating

	Is case definition adequate?	Representativeness of cases	Selection of controls	Definition of controls
**Possible answers**<br><br>**(bold = answer for study)**	**a) yes, with independent validation: ***<br>b) yes, eg record linkage or based on self reports<br>c) no description	**a) consecutive or obviously representative series of cases: ***<br>b) potential for selection biases or not stated	**a) community controls: ***<br>b) hospital controls<br>c) no description	**a) no history of disease (endpoint): ***<br>b) no description of source
**Applied to study**	Cases were infants with severe IVH, clearly defined as grade 3 or 4 IVH or peri-ventricular hemorrhagic infarction, using standard classification system (Papile classification, cranial ultrasound).	The used data from the population-based EXPRESS cohort, consisting of infants with GA of 22+0days to 26wks+6 days, born from 1 April 2004 to 31 March 2007 in Sweden. Ensuring a representative series of cases of ELBW infants with severe IVH.	Controls were matched for gestational age, birth weight, sex, and hospital with the cases, and taken from the same cohort as the cases.	Controls were specifically defined as infants without IVH (by cranial ultrasound).

Comparability: 2 stars rating

	Comparability of cases and controls on the basis of the design or analysis
**Possible answers (bold = answer for study)**	**a) study controls for ### (Select the most important factor.) : ***<br>**b) study controls for any additional factor: * (This criteria could be modified to indicate specific control for a second important factor.)**
**Applied to study**	Matching based on critical factors as GA, BW and sex. Furthermore, statistical adjustments were made for other potential cofounders such as mechanical ventilation and CRIB score.

Exposure: 3 stars rating

	Ascertainment of exposure	Same method of ascertainment for cases and controls?	Non-Response rate
**Possible answers**<br><br>**(bold = answer for study)**	**a) secure record: ***<br>b) structured interview where blind to case/control status: *<br>c) interview not blinded to case/control status<br>d) written self report or medical record only<br>e) no description	**a) yes: ***<br>b) no	**a) same rate for both groups: ***<br>b) non respondents described<br>c) rate different and no designation
**Applied to study**	Medical records	The method of ascertaining exposure was the same for both the cases and controls, as the data was retrieved from hospital records.	No significant non-response mentioned.

Quality rating: 9/9 stars = **High quality.**

The case and control groups were well-defined, and the exposure was accurately measured using reliable methods. There were adjustments for potential confounders, and the study design was appropriate for answering the research question. The risk of bias is minimal, with careful matching and use of validated classification systems.

Limitations: retrospective study.

Case reports (2):

1. **"Severe hypernatraemia in a very preterm infant"** by Saeed, M.A.[10]

Tool = JBI Checklist Evaluation:

**Criteria**	**Yes**	**No**	**Unclear/ not applicable**	**Notes**
**Were patient's demographic characteristics clearly described?**	X			- Information about age, sex, birth weight, mothers' health, previous and current treatment and diagnostic test results and medications are available.<br>- No information about race.
**Was the patient's history clearly described and presented as a timeline?**		X		- There is information on delivery mode, initial complications and interventions after birth, clearly described in time.<br>- There is no detailed information about course of the pregnancy.<br>- Information on clinical condition at birth is not well described, e.g no Apgar scores are reported.
**Was the current clinical condition of the patient on presentation clearly described?**	X			- Clear course of case after birth until occurrence of hypernatremia is given.<br>- Information on condition when hypernatremia occurred is given.
**Were diagnostic tests or assessment methods and the results clearly described?**	X			- Diagnostic tests including serum sodium levels, other laboratory findings and estimation of urine output is described.<br>- Sodium concentration and weight change during first 96h is shown in a figure.
**Was the intervention(s) or treatment procedure(s) clearly described?**	X			- There is a clear description of all the treatments/interventions given in time.<br>- Fluid intake during first 96h (type and quantity) is shown in a figure.
**Was the post-intervention clinical condition clearly described?**	X			- Clear description of clinical course after treatment was given: evolution of sodium, urine output, creatinine,…<br>- Description of recorvery and neurodevelopmental outcomes at 6 months, 1 year, and 2 years is given.<br>- Neurodevelopment was measured with Denver scale.<br>- No information on imaging available
**Were adverse events (harms) or unanticipated events identified and described?**			X	- No major adverse events were noted, and the infant was successfully treated without long-term morbidity
**Does the case report provide takeaway lessons?**	X			- Information of possible cause of hypernatremia in ELBW infants is given.<br>- Valuable lessons about the management of severe hypernatraemia in preterm infants, particularly regarding fluid management

				and the need for close monitoring in similar cases are provided.

Overall appraisal: Include article, **moderate quality.**
The case report meets most of the criteria of JBI critical appraisal. The report is well-documented, and the methodology is sound. It provides useful information for clinical practice.

Limitations case report in general:
In general case reports are low evidence. While they can provide valuable insights case reports have several limitations. Some of the limitations may be:

- Lack of generalizability: focus on a single patient, findings are not representative of the broader population. The results cannot be generalized to other individuals or settings.
- No control group for comparison, so there is no baseline against which the outcomes can be compared. Difficult to determine whether observed outcomes are due to the exposure, intervention, treatment of other factors not controlled for.
- Subject to bias: single cases can be influenced by various types of bias e.g selection bias, the patient chosen may not be representative of the broader population,…
- Causality is not established.
- No statistical analysis, no way to assess the strength of an association.
- No systemic follow-up. The lack of follow-up data limits the ability to assess the full impact of the exposure, treatment or intervention over time.
- Often reporting of a rare or extreme event. findings might not be representative of typical clinical practice or more common conditions.
- Incomplete reporting. They may lack certain information, depending on what the author chose to highlight, leading to difficulty in interpreting the findings or reproducing the results.

For broader conclusions, more robust study designs like cohort studies or randomized controlled trials are required.

2. "**Severe Hypernatremia in an Extremely Low Birth Weight Infant with Subsequent Normal Neurological Development",** by Sabir et al. [13]

JBI Checklist Evaluation:

**Criteria**	**Yes**	**No**	**Unclear/ not applicabl e**	**Notes**
**Were patient's demographic characteristics clearly described?**	X			- Information on age, sex, BW, length, head circumference, previous and current treatment and diagnostic test results and medications are available.<br>- No information about race, mothers' health.
**Was the patient's history clearly described and presented as a timeline?**	X			- A clear description of course of pregnancy (complications), mode of delivery, early complications after birth and prenatal care/early treatments and interventions is given in time.
**Was the current clinical condition of the patient on presentation clearly described?**	X			- A clear course of birth until occurrence of hypernatremia is given.<br>- The condition of the case when hypernatremia occurred is well described.
**Were diagnostic tests or assessment methods and the results clearly described?**	X			- Diagnostic tests including serum sodium levels, ultrasonography, and blood gas analysis were clearly described<br>- Overview of sodium concentrations over time are provided in a figure.
**Was the intervention(s) or treatment**	X			- Detailed description of treatment protocols, including fluid management given (type, quantity) in time.

**procedure(s) clearly described?**				
**Was the post-intervention clinical condition clearly described?**	X			- Clear description of clinical course after treatment was given: evolution of sodium, urine output, blood glucose, capillary blood gases,…<br>- Cerebral ultrasound data is provided.<br>- Neurodevelopmental evaluation at 4 months corrected age by MRI, and 6 months corrected age by Bayley scales of Infant development-II
**Were adverse events (harms) or unanticipated events identified and described?**			X	- Article does not present any significant adverse neurological (or other) outcomes despite the severe sodium imbalance
**Does the case report provide takeaway lessons?**	X			- Cause of sodium imbalance is given.<br>- The case provides key lessons regarding the management of hypernatremia in preterm infants, and the need for precise fluid management.

Overall appraisal: Include, **high quality** case report:
The case meets the criteria for JBI critical appraisal. The report is thorough, and the treatment and outcomes are well-documented. It provides useful information and lessons for clinicians

Case reports in general: low evidence, see higher.
