# Supplementary material for: Sodium Values During the First 10 Postnatal Days in Extremely-Low-Birth-Weight Infants and Long-Term Neurocognitive Outcomes: A Systematic Review
Source: Children (Basel). 2026 Feb 19;13(2):287. doi: 10.3390/children13020287 (PMC12939160; doi:10.3390/children13020287)
Supplement: Supplementary file 1 [file children-13-00287-s001.zip › Table S1.pdf]

Table S1: Search strategy used in PubMed, Embase, Web of Science Core Collection, CINAHL, and Scopus

**Database**	**Search string**
**Pubmed (including medline)**	("Infant, Low Birth Weight"[Mesh] OR "Low Birth Weight*"[tiab] OR "Low Birthweight*"[tiab] OR "LBW"[tiab] OR "VLBW"[tiab] OR "ELBW"[tiab] OR "Infant, Premature"[Mesh] OR "preterm"[tiab] OR "premature*"[tiab] OR "prematurity"[tiab]) AND ("Hypernatremia"[Mesh] OR "Hyponatremia"[Mesh] OR "Sodium"[Mesh] OR "Hypernatr*"[tiab] OR "Hyponatr*"[tiab] OR "Sodium"[tiab] OR "Dysnatr*"[tiab] OR "Salt" [tiab])
**Embase (via Embase.com)**<br><br>Science Citation Index Expanded (SCI-EXPANDED)--1955-present Social Sciences Citation Index (SSCI)--1956- present<br>Arts & Humanities Citation Index (AHCI)--1975-present Conference Proceedings Citation Index – Science (CPCI-S)--1990-present Conference Proceedings Citation Index – Social Science & Humanities (CPCI-SSH)- -1990-present Emerging Sources Citation Index (ESCI)-- 2019-present	('low birth weight'/exp OR 'low birth weight*':ti,ab,kw OR 'low birthweight*':ti,ab,kw OR 'prematurity'/exp OR 'preterm':ti,ab,kw OR 'premature*':ti,ab,kw OR 'prematurity':ti,ab,kw OR 'ELBW':ti,ab,kw OR 'VLBW':ti,ab,kw OR 'LBW':ti,ab,kw) AND ('sodium'/exp OR 'sodium':ti,ab,kw OR 'hypernatremia'/exp OR 'hypernatr*':ti,ab,kw OR 'hyponatremia'/exp OR 'hyponatr*':ti,ab,kw OR 'dysnatremia'/exp OR 'dysnatr*':ti,ab,kw OR 'salt':ti,ab,kw) NOT 'conference abstract':it
**WoS Core Collection**	(TS=("low birth weight" OR "low birth weight*" OR "low birthweight*" OR "LBW" OR "VLBW" OR "ELBW" OR "prematurity" OR "Premature*" OR "Preterm")) AND (TS=("Hypernatr*" OR "Hyponatr*" OR "Sodium" OR "Dysnatr*" OR "Salt")) NOT (DT=("meeting abstract"))
**CINAHL (via EBSCOhost)**	((MH "Infant, Low Birth Weight+") OR (MH "Infant, Premature") OR TI ("Low Birth Weight" OR "Low Birth Weight*" OR "Low Birthweight*" OR "ELBW" OR "VLBW" OR "LBW" OR "preterm" OR "premature*" OR "prematurity") OR AB ("Low Birth Weight" OR "Low Birth Weight*" OR "Low Birthweight*" OR "ELBW" OR "VLBW" OR "LBW" OR "preterm" OR "premature*" OR "prematurity")) AND ((MH "Hypernatremia") OR (MH "Sodium") OR (MH "Electrolyte Management: Hypernatremia (Iowa NIC)") OR (MH "Hyponatremia") OR (MH "Electrolyte Management: Hyponatremia (Iowa NIC)") OR TI("sodium" OR "hypernatr*" OR "hyponatr*" OR "dysnatr*" OR "salt") OR AB("sodium" OR "hypernatr*" OR "hyponatr*" OR "dysnatr*" OR "salt"))
**Scopus**<br><br>(limit to articles and reviews)	(TITLE-ABS("low birth weight*" OR "low birthweight*" OR "LBW" OR "VLBW" OR "ELBW" OR "prematurity" OR "Premature" OR "Preterm")) AND (TITLE-ABS("sodium" OR "hypernatr*" OR "hyponatr*" OR "dysnatr*" OR "salt"))
